# Conspiracy spillovers and geoengineering

**DOI:** 10.1016/j.isci.2023.106166

**Published:** 2023-02-28

**Authors:** Ramit Debnath, David M. Reiner, Benjamin K. Sovacool, Finn Müller-Hansen, Tim Repke, R. Michael Alvarez, Shaun D. Fitzgerald

**Affiliations:** 1 Cambridge Zero, University of Cambridge, Cambridge CB3 0HE, UK; 2 Division of Humanities and Social Science, California Institute of Technology, Pasadena, CA 91125, USA; 3 Centre for Climate Repair, Department of Engineering, University of Cambridge, Cambridge CB2 1PZ, UK; 4 Energy Policy Research Group, Judge Business School, University of Cambridge, Cambridge CB2 1AG, UK; 5 University of Sussex Business School, Brighton BN1 9SN, UK; 6 Institute for Global Sustainability, Boston University, Boston, MA 02215, USA; 7 Department of Business Development and Technology, Aarhus University, 7400 Herning, Denmark; 8 Mercator Research Institute on Global Commons and Climate Change, 10829 Berlin, Germany

**Keywords:** Engineering, Energy engineering, Social sciences, Research methodology social sciences

## Abstract

Geoengineering techniques such as solar radiation management (SRM) could be part of a future technology portfolio to limit global temperature change. However, there is public opposition to research and deployment of SRM technologies. We use 814,924 English-language tweets containing #geoengineering globally over 13 years (2009–2021) to explore public emotions, perceptions, and attitudes toward SRM using natural language processing, deep learning, and network analysis. We find that specific conspiracy theories influence public reactions toward geoengineering, especially regarding “chemtrails” (whereby airplanes allegedly spray poison or modify weather through contrails). Furthermore, conspiracies tend to spillover, shaping regional debates in the UK, USA, India, and Sweden and connecting with broader political considerations. We also find that positive emotions rise on both the global and country scales following events related to SRM governance, and negative and neutral emotions increase following SRM projects and announcements of experiments. Finally, we also find that online toxicity shapes the breadth of spillover effects, further influencing anti-SRM views.

## Highlights

• Social media users react to new climate technologies like solar geoengineering (SG)
• Deep learning and natural language processing measured online discourse's toxicity
• Conspiracy theories like chemtrails affect online public negativity related to SG
• SG conspiracies use UK, US, India, and Sweden geopolitics

## Graphical abstract

**Figure:**
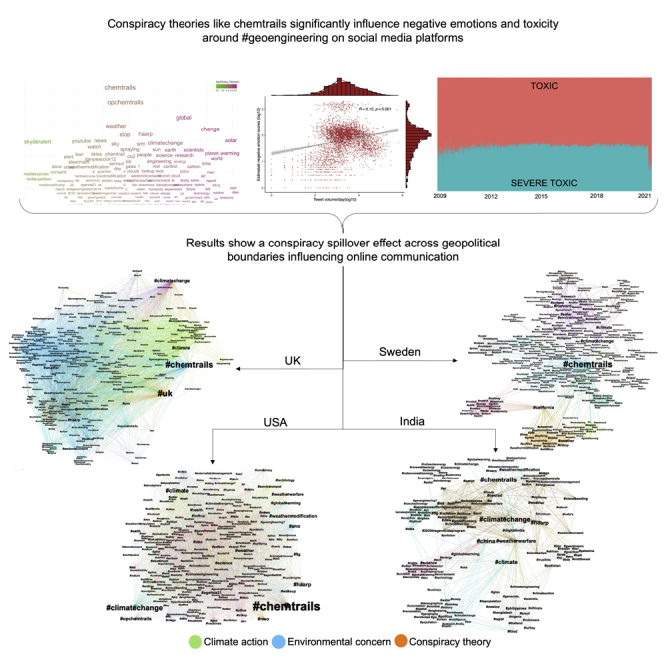

## Introduction

As the calls for climate action intensify,^1^^,^^2^ climate engineering technologies, in particular solar radiation management (SRM), have received increasing attention, and public controversy has ensued. SRM includes technologies such as space-based shields, stratospheric aerosols, cirrus cloud thinning, marine cloud brightening, and increasing surface albedo.^3^^,^^4^^,^^5^ While the broader conception of geoengineering may also include greenhouse gas removal options (such as large-scale afforestation or direct air capture and storage), most geoengineering debates focus on “solar geoengineering (SG)”, often referred to as solar radiation management or solar radiation modification (SRM).

There is opposition to both SG research and SG deployment. Opposition to SG research arises from not just concerns about its potential methods (which are still hypothetical), and potential harms,^6^ but the uncertainties and risks associated with the path from research to problems after deployment, which are politically expensive.^4^^,^^6^ Outdoor SG experiments have already resulted in sustained opposition from civil society and indigenous groups.^7^ Concerns have reached a level where some have argued for a global treaty prohibiting research, deployment, and use.^8^ Calls to block SG research highlight the need to explore what specific issues different groups are raising about SG and the mechanism(s) by which these concerns are disseminated.

Concerns include the potential weaponization of weather control, attribution of weather-related disasters to an SG program, deepening of conflict from perceived illegitimacy of its deployment across sensitive geopolitical areas,^4^^,^^9^^,^^10^^,^^11^^,^^12^ as well as temptations for SG to be used to tailor the climate for a single country’s benefit,^10^^,^^13^ including redistribution of health risks and income inequality.^14^^,^^15^ Therefore, many experts call for more research to better understand SG.^3^ Others have urged social scientists to help inform the design of future research.^16^ For example, a 2021 US National Academies of Sciences, Engineering, and Medicine (NASEM) report^3^ emphasized the need to better understand the dichotomy of uncertainties and risks associated with SG research. The report concluded that SG research should include public programs for accountability and called for more robust research governance, including scale limits on field experiments, periodic program reassessment, and broader international cooperation.^3^^,^^6^^,^^17^

Other concerns focus on the politics and ethics of SG. Efforts to integrate Global South actors into SRM initiatives remain limited.^18^^,^^19^ Also, the development of climate engineering options often fails to tap into truly inclusive or responsible forms of innovation or technology design.^20^^,^^21^^,^^22^

The concerns and criticisms of SG discussed above largely reflect expert opinion rather than public perceptions and attitudes toward SG. This paper seeks to help address this knowledge gap by analyzing historical and social media interactions concerning various global SG events and investigating potential correlations with other controversial topics, such as conspiracy theories.

Recent studies have shown that conspiracy theories, propelled by fake news and polarization (i.e. disinformation campaigns), can act as a contagion of intense negative emotions in digital social networks.^23^^,^^24^^,^^25^ Disinformation campaigns can have a pernicious and long-lasting negative impact on beliefs about climate change and on public acceptance of more sustainable lifestyles. For example, misinformation campaigns on social media in Brazil condoned and then perpetuated renewed deforestation of the Amazon rainforest.^26^ In Europe, false conspiracy theories were spread (and believed), connecting plans to build a new gas pipeline with the mafia’s involvement and the spread of infectious diseases.^27^ One patently false conspiracy theory dating back to the mid-1990s is “chemtrails” (explored in greater depth later), which alleges condensation trails (contrails) from aircraft are intentionally seeded with chemical or biological compounds for various nefarious purposes such as population control, military testing or, most relevant to our case, weather and climate modification including SG. Up to 40% of Americans believe this theory to be “somewhat true”, which has influenced social attitudes about climate policy, and geoengineering.^25^

On climate change, there is growing evidence of a “spillover” effect that leverages local conflict/controversies to cascade controversies in order to shift a policy agenda deliberately^28^^,^^29^^,^^30^, similar to conventional agenda-setting. In this paper, we expand on the conceptual application of the “spillover effect” to evaluate conspiracy theories across geopolitical boundaries and their agenda-setting impact on public emotions and online toxicity perceptions of SG research.

This paper is distinct from previous studies critiquing SG governance challenges and their associated controversies related to climate action, on which there is already a rich literature,^3^^,^^4^^,^^6^^,^^7^^,^^16^^,^^25^^,^^31^^,^^32^^,^^33^ including social media mining-based SG conspiracy analysis.^25^ One apparent gap in Tingley and Wagner’s^25^ study is that the authors had a narrow focus on the “chemtrails” conspiracy theory in their searches for data collection. Therefore, they could not measure any spillover effect from other conspiracy theories in geoengineering debates.

This study uses digital data from social media to capture cross-sectional variation in public emotions following major SG announcements on a global and country scale (UK, USA, Sweden, and India) using geospecific hashtags. At the same time, we investigate whether social media is used to propagate and influence conspiracy theories on SG, thereby deepening our understanding of potential spillovers of controversial research topics into the public domain. Themes and ideas condensed in hashtags spread between user groups and regions in ways that are influenced by other themes and beliefs attached to the same tweets. Hence, as a result of “conspiracy spillovers”, we find that the online communication of SG associated with conspiracy theories spreads by taking advantage of the geopolitical controversies.

Our scope is limited to using social media interaction data as a proxy for cross-sectional public opinion on geoengineering. It may be non-representative of the broader public opinion. However, recent studies have shown that it is legitimate to say that social media is a breeding ground for conspiracy theories and hence deserving of attention.^34^ Specifically, Twitter-led climate communication is increasingly seen to engage its user toward climate action^35^^,^^36^^,^^37^ using tools like hashtags.

Users on Twitter employ hashtags to indicate the general theme or topic they wish to associate with their tweets. This allows one to investigate how information propagates throughout the platform. As hashtags show an attention-seeking behavior, it makes them a viable candidate^38^ for studying the public reaction to complex governance issues of SG and its interface with conspiracy theories. We use natural language processing, deep learning, and network analysis (see STAR Methods) to explore users’ reactions in 814,924 English-language tweets containing #geoengineering across 13 years (January 2009–November 2021).

## Results

### Public emotion on social media shifts following SG announcements

This section presents three longitudinal results based on the natural language processing (NLP) and lexicon-based emotional analysis of the #geoengineering Twitter dataset. Here, we establish the dynamic relationship between the user reactions to major solar geoengineering (SG) projects and governance events in the public domain.

**Figure 1:**
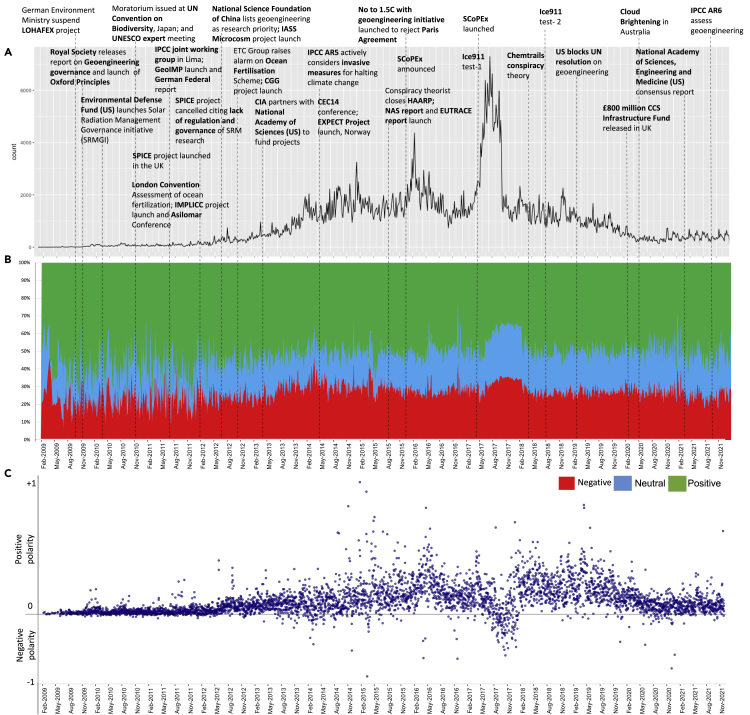
Online emotions to #geoengineering (A) Time-series representation of the number of tweets on #geoengineering and major global SG project and governance events (2009–2021). (B) 6-month moving average estimates of emotion shares in the tweets (n = 814,924). [Note: The emotion scores are estimated using a lexicon-based approach (NRC-lexicon) with the following embedded emotions in each category: positive emotion (joy, trust, and optimism), negative emotion (disgust, sadness, fear, and anger), and neutral emotion (anticipation and surprise), (see STAR Methods section for more detail)]. (C) Emotion polarity plot of the time-series tweets [Note: polarity estimated as positive minus negative emotions].

The first finding is a strong relationship between Twitter activity concerning major SG projects and climate action or negotiation events (see Figure 1A). For example, the number of #geoengineering tweets increased around the launch of the EXPECT project in Norway and when the IPCC actively considered invasive measures to halt climate change in May 2014. A significant rise in Twitter activity was also observed following reporting about the SCoPEx (Stratospheric Controlled Perturbation Experiment) project, led by David Keith and colleagues at Harvard University and supported by a consortium of funders that also included Bill Gates, a long-standing favorite target of conspiracy theorists. The project was announced in December 2015, and SG protests were held at COP21 in Paris (Figure 1A). Twitter volume on #geoengineering peaked when the SCoPeX project was officially launched in April 2017. This was the most significant surge in Twitter activity on #geoengineering, with close to 8,000 interactions per day (an ∼ 300% increase relative to February 2017 levels) in the peak period (June 2017, Figure 1A). Twitter activity returned to pre-SCoPEx levels by August 2017, followed by new but smaller peaks in the chemtrails conspiracy-related online interactions. We also see from Figure 1 that “opchemtrails” topics disappeared toward the end of December 2017.

The trendline in Figure 1A can be divided into two phases of SG project development. Pre-2016, there were more governance-related actions involving various multilateral organizations. However, post-2016, significant announcements were made regarding practical implementation pilot projects and experiments.

Secondly, the moving-average emotion analysis in Figure 1B shows that positive emotions (i.e. optimism, joy, and trust) are more prominent, recurrent, and comprise a larger share of the #geoengineering social media conversation. The share of negative (i.e., disgust, sadness, fear, and anger) and neutral (i.e., anticipation and surprise) emotions increase over time, with distinct peaks between June 2017 and April 2018 (Figure 1B). We also see that emotions are more volatile in the 2009–2012 period, with short-lived spikes in the share of negative (rising from 15% to 30%) and neutral (24.5%–40%) emotions following governance-related events such as the publication of the Royal Society report and the Oxford Principles both in 2009 (Figure 1B). A similar increase in emotion volatility was observed in late 2010 (positive emotions increased from 40% to 55%), which coincided with the launch of the SPICE project and calls for a moratorium on SG technologies at the UN Convention on Biodiversity citing gaps in scientific knowledge and risk management.^39^ The share of negative and neutral emotions also spiked during the cancellation of the field-trial aspects of the SPICE project in January 2012 (see Figure 1B).

**Figure 2:**
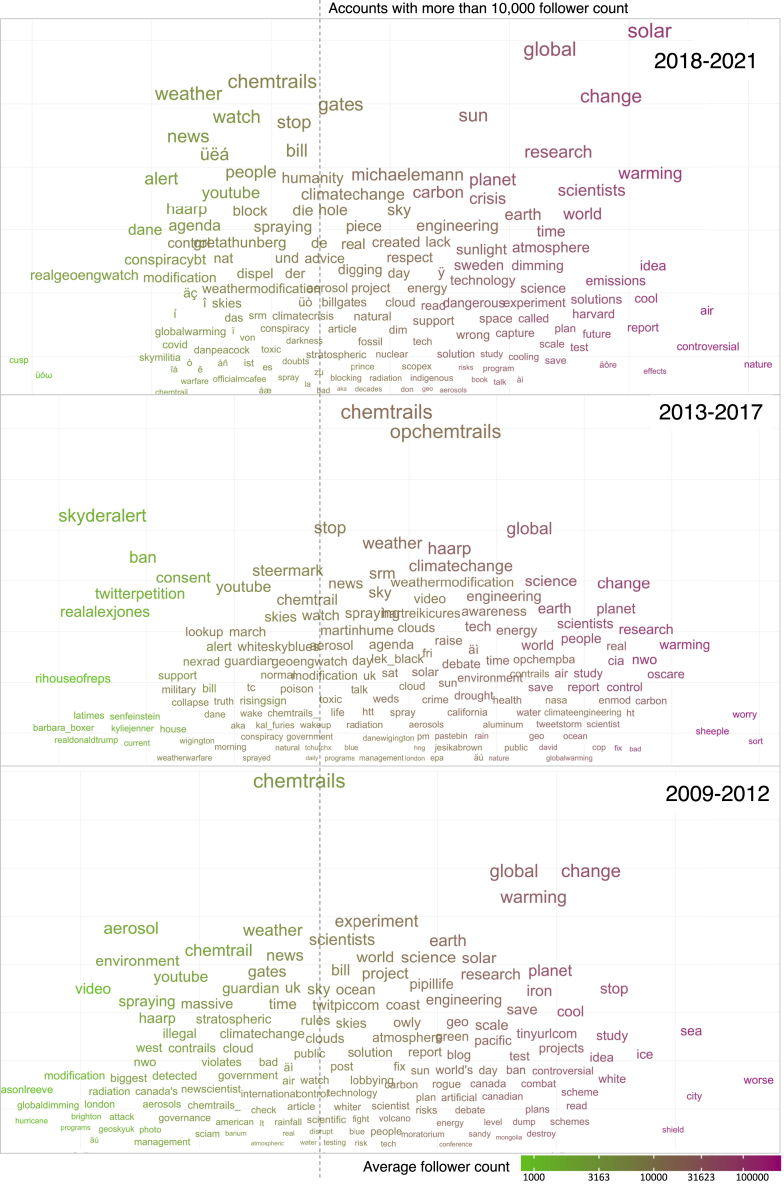
A chatter plot demonstrating the spatial distribution of Twitter engagement on #geoengineering and its semantic structure based on three time periods The size of the words/terms corresponds to their occurrence in the Tweet corpus (n = 814,924), here termed “frequency”. The color of the words represents the average follower base of the users. We considered a user highly influential if its average follower count is at least 10,000, shown by a black dashed line. [Note: Some of these highly influential accounts may be bots, and the actual human influencers’ follower base can differ].

**Figure 3:**
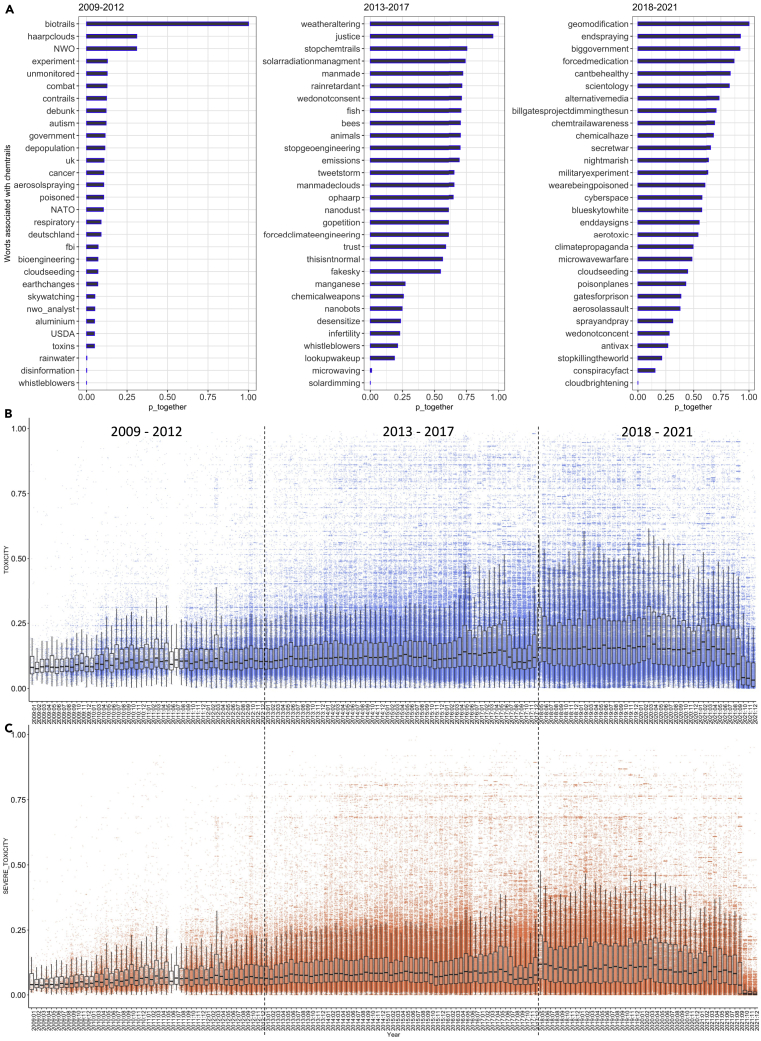
Word embeddings and online toxicity (A) Words associated with “chemtrails” conspiracy theory from word embeddings of the #geoengineering debate on Twitter for three distinct periods. The x axis shows the conditional probability of association (p_together) of specific neighboring words with “chemtrails” in the tweet corpus (n = 814,924). (B and C) toxicity score, and (c) Severe toxicity score of the tweets across the timescale, illustrated as time-series boxplots. The y axis is a normalized scoring scale between 0 and 1, and the x axis represents discrete year and month (Year:Month).

Moving averages in Figure 1B (2018–2021) show public emotions possibly modulated by conspiracy theories like HAARP (an ionospheric research program largely funded by the US military) and chemtrails. Later in Figures 2 and 3A, we show the broadening of conspiracy theories to include the Covid-related “antivaxx” movement (late 2021) through the semantic structure of the online conversation. In line with findings in the literature,^25^^,^^40^^,^^41^^,^^42^ this shows the possibility that there are spillover effects between different conspiracy theories. We also found increases in shares of negative (from 20.4% to 28.6%) and neutral (from 24.3% to 47.7%) emotions following SG-related field experiments (Figure 1B). The share of neutral emotions, in particular, grew substantially following attempted SG field experiments. In contrast, negative emotions rose following announcements of these projects.

The 2021 NASEM consensus report was critical for policy debates, emphasizing that SG research should be cataloged in a public registry and research on social dimensions of SG should be prioritized.^3^ When this report was released, which outlined the type of research that should be conducted, how it should be governed, and who should fund it, the most specific emotion change was the rise in neutral and positive emotions (Figure 1B).

Third, increased social media engagement in geoengineering is associated with prominent shifts in online emotion trends. For example, we show in Figure 1C that with an increase in Twitter volume from April 2017 to May 2018, the emotional polarity of the tweets reverses from predominantly positive to predominantly negative. It was more notable during the controversial SCoPEx project launch. Our results show that emotion polarity is significantly reversed from positive to negative following the launch of SG projects; the rise in negative emotions is statistically significant ($R^{2}=10.02$, 99% CI) (Figure S1). However, following the announcement of governance-based actions, the polarity reverses from negative to predominantly positive (see Figure 1C).

### Conspiracy theories become more prevalent in online communication over time

This section presents the results of a dynamic semantic analysis of the tweets over the 13-year time period using NLP, word embedding identification, and deep learning-based online toxicity analysis. For ease of interpretation, we categorize this time period into three time frames, 2009–2012, 2013–2017, and 2018–2021.

A distinct semantic finding in the tweets is an extreme occurrence of the term “chemtrails” (see Figure 2), which is a conspiracy theory.^25^ This term becomes more prevalent in tweets (see Figure 2). We find that between 2009 and 2012, influential users engaged with words such as “scientists”, “experiment”, “lobbying”, “controversial”, “ban”, “worse”, etc., in high frequency (i.e., used these words at least 1000 times per year in their tweets). Words and associated terms like “weathermodification”, “contrails”, “government”, and “haarp” were also shared in a similar frequency, implying that there is online communication on the science but also on conspiracy-related factors (see Figure 2). Users with a reasonable number of followers (sim 3000–10,000) shared conspiracy-related terms, indicating that Twitter users are shaping an overall anti-geoengineering narrative, notably supported by accounts with high follower counts (> 10,000). Interestingly, “chemtrails” had the highest frequency (> 10,000) of tweets, prevalent among highly influential users.

To better understand the role of conspiracy theories in SG discussions, we analyzed the semantic structure of the chemtrails conspiracy theory using word embeddings (Figure 3). For example, between 2009 and 2012, words and topics having the highest probability (p_together) of being associated with chemtrails included SG experiments and concerns around its perceived impact. From 2013 to 2017, high probability words indicated a greater association of tweets opposed to SG experiments by leveraging chemtrails conspiracies. Finally, over 2018 to 2021, the associated terms/hashtags primarily characterize how people perceive the alleged spraying or demand action to stop it (Figure 3A).

Our results also show that the chemtrails conspiracy became semantically richer from 2018, as it began to piggyback on climate action and justice wording (see Figure 3A, details in Figure S4). This broadening also targeted the engagement around anti-SG narratives on weaponization and depopulation (supporting findings of^25^). Another important finding about the dynamics of chemtrails over time is that tweets associated with them became increasingly toxic. The toxicity attributes are shown in Figures 3B and 3C. The mean toxicity score is 0.17 and the severe toxicity score is 0.12 (details can be found in Table S2 and Figure S2). We also see an increase in median toxicity attributes scores for 2018–21, which can be associated with increasing Twitter engagement (see Figure S2C). The tweets with the highest toxicity scores during this period are illustrated in Table 1, demonstrating the prevalence of strong anti-SG and chemtrails online narratives.

**Table 1:** Tweets with highest toxicity scores across the 13-year time frame

Toxicity score	Time frame	Original Tweet
0.989	2018–21	Go f∗∗k yourself s∗∗tty ex basketball player with stupid takes. Take a trip down the geoengineering lane and quit being a dumb a∗∗
0.987	2018–21	f∗∗k me, geoengineering advocates be stupid af F∗∗k bill gates in the god damn a∗∗hole. Geoengineering our way out of climate change is the most dangerous s∗∗t ive ever heard. The danger of putting this in the atmosphere is a hellscape. Once you start spraying it, you can never stop. It damns all life on planet earth
0.978	2018–21	F∗∗k bill gates in the god damn a∗∗hole. Geoengineering our way out of climate change is the most dangerous s∗∗t
0.959	2018–21	lmfao this s∗∗t sucks so much. geoengineering is starting to sound reasonable. and that’s a terrible idea. Global Geoengineering? Get the f∗∗k out of here, you pseudoscience moron! Keep your stupid vaccines and your lame ass geoengineering. crappy geoengineering rainbow - more and more common. look at that sh∗∗∗y white out sky … sigh. b∗∗∗∗∗∗s. Who in the F∗∗K teaches geoengineering and ethical hacking?, Why is it not APPARENT you are criminals??
0.989	2013–17	F∗∗k u all so bad- hope u die the worst death ever #geoengineering daily poison @POTUS @FBI @CIA @NSAGov @NoradNorthcom @USNavy @usairforce Climate engineering sucks!! #GeoEngineering,F∗∗K YOU #HurricanePatricia!! #AnimoMexico!!
0.987	2013–17	F∗∗k geoengineering f∗∗k the government f∗∗k the system f∗∗k haarp F∗∗k weather warfare f∗∗k chemtrails f∗∗k those shoes F∗∗k your haircut F∗∗k #FIFA. Wake up, a∗∗holes. U care more about sports than u do #Geoengineering #Fukishima #GMO or #Reality. F∗∗k #HBO #GameofThrones #chemtrails #Geoengineering read the F∗∗king #books I don’t care if you don’t like me tweeting this s∗∗t ! ITS F∗∗kING UP YOUR PLANET TOO !#geoengineering #sag #srm
0.979	2013–17	Warning: Explicit Language Alert F∗∗k your planes, F∗∗k your chemicals, F∗∗k your Geoengineering, F∗∗k your lines in the sky, and F∗∗k you STOP SPRAYING POISONS at least on EARTH DAY, you a∗∗holes #chemtrails #haarp #geoengineering #noaa #nasa #climate #faa #epa #fda #cnn I’m so goddamn sad you sick F∗∗k geoengineering earth making fires burn hotter sick sick F∗∗ks nazi stop chemtrails
0.967	2013–17	Call me a conspiracy theorist for believing in geoengineering pisses me off. F∗∗king sheeple. F∗∗king #Chemtrails From F∗∗king #Geoengineering And F∗∗king #Nexrad. NOT F∗∗kING ANGELS IN THE SKY FROM F∗∗kING GOD. STOP PLAYING GOD YOU F∗∗kING BASTARDS! #GMO #chemtrails #geoengineering #illuminati #NWO #Monsanto
0.959	2013–17	We are getting creamed in Bristol ! LOOK THE F∗∗k UP #bristol #geoengineering This is getting stupid Massive attack in Bristol, uk right now !not the F∗∗king band you morons#geoengineering F∗∗k war propagandists. F∗∗k Hugh Grant. F∗∗k glyphosate Roundup. F∗∗k geoengineering. F∗∗k Horacio Rozanski. F∗∗k Ptech. F∗∗k corporate interests F∗∗k THE IPCC AND THEIR GEOENGINEERING! CLIMATE CHANGE HAS NOTHING TO DO WITH Co2 ! Special Report: Geoengineering Dumbing Down America, Not Carbon Dioxide @LeoDiCaprio YOU ARE A GLOBALIST IDIOT! WATCH! THIS IS THE REASON 4 CLIMATE CHANGE. SPEAK OUT ABOUT GEOENGINEERING!!
0.989	2009–12	Why is Bill Gates pouring millions into geoengineering? because he’s a F∗∗king predator. Good Grief that crazy B∗∗∗h Cynthia McKinney is talking at a geoengineering conference. Anything for money. What a b∗∗∗h.anyone who is so stupid they don’t know about geoengineering, check out the sky above wales today. cu∗∗s. F∗∗k HAARP. F∗∗k their manmade earthquakes & geoengineering. LOOK AT WHAT THEY HAVE DONE.
0.967	2009–12	F∗∗k off Defra! UK smog warning my arse, u mean planes are coming to spray the sky with chemicals # Chemtrails #HAARP #geoengineering F∗∗kING MOTHER F∗∗kERS!!! KMT!! BBC ADMIT TO AEROSOL SPRAYING/CHEMTRAILS GEOENGINEERING Obamas white house science czar John holdren ADMITS they are geoengineering the planet! That explains chemtrail bombardment! F∗∗k YOU!
0.959	2009–12	the real story is nor climate change - it’s geoengineering of the planet but you are a stupid human(liberal) it doesn’t matter 2u @BillGates what’s with the #Geoengineering #PopulationControl you dumb ass geek.. your stolen operating system sucks ass so do you The sky right above my neighborhood is crisscrossed with #chemtrails. F∗∗k you #geoengineering
0.950	2009–12	saw plenty of chemtrails on my drive home sooooooooo …. F∗∗k the system. F∗∗k THEIR geoengineering. F∗∗k the government & the elitists F∗∗k al gore for propagating the LIE that is “global warming” and F∗∗k HAARP & their GEOENGINEERING + weather manipulation
0.940	2009–12	sorry U feel that way! When u decide to get your head out your ass! Research Geoengineering! Its no conspiracy its scientific fact sky is just covered in chemtrails today. sickening. #F∗∗kHAARP & their high altitude low frequency weapons #geoengineering until they stop geoengineering/creating earthquakes in places like haiti and Japan, i will always say F∗∗k HAARP. OUR ATMOSPHERE IS PRECIOUS, WHY DO WE F∗∗k IT UP LIKE THIS???? #Chemtrails #Geoengineering #SRM #Weather #SkyWatching

Note: ∗ added by the authors to mask explicit language.

We find a significant correlation ($R^{2}=8.12$, 99% CI) between an increase in daily Twitter volume on SG and an increase in toxicity scores. A similar significant correlation was also observed for severe toxicity scores ($R^{2}=7.19$, 99% CI) (shown in Figure S2C). Thus, greater Twitter engagement on SG-related topics is associated with more negative emotions.

**Figure 4:**
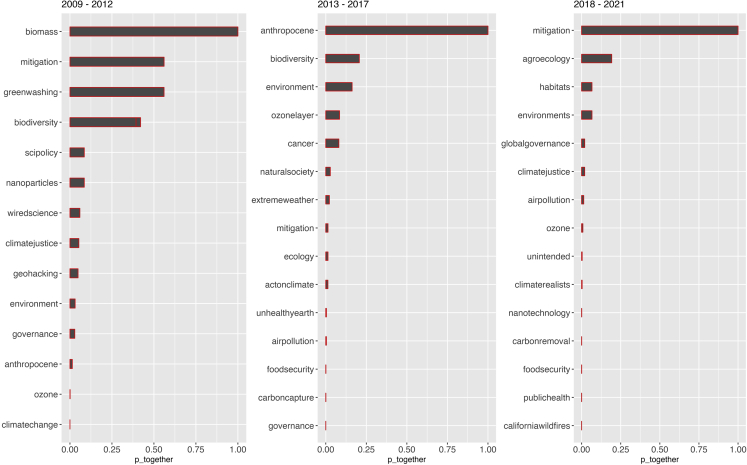
Words associated with “geoengineering” obtained through its word embedding estimations over the three periods Here, we filtered the chemtrails from the corpus to evaluate the semantic structure of non-conspiracy #geoengineering interactions in the tweet corpus. The x axis shows the probability score (0–1) of the word embeddings of “geoengineering”.

There are, of course, many legitimate concerns raised by environmental scientists or others concerned about the deployment of geoengineering. When we evaluate the associated word with geoengineering and filter chemtrails-related terms, the results show evolving use of terms like “mitigation”, “biodiversity”, “airpollution”, “ozone”, “environment”, and so on (see Figure 4). We found these terms are less frequent in the tweet corpus than conspiracy-related terms, as shown in Figure 2. Their semantic existence implies the presence of an online discourse on the environmental dimensions of geoengineering, like its unintended impact on biodiversity, climate justice, ecology, food security, and public health. However, how these discussions influence conspiracy theories are beyond this paper’s scope.

### Online conspiracy spillover influences geospecific SG agenda

This section expands on the previous results regarding the influence of conspiracy theories in driving user engagement and shaping anti-SG-narratives on Twitter. Here, we show the presence of a spillover effect of the chemtrail conspiracy theory across geographical boundaries using network theory. In addition, we evaluate the influence of such spillover on online toxicity geospatially.

Current evidence shows that concerns over SG research, deployment, and governance transcend geopolitical boundaries.^20^^,^^21^^,^^31^ Here, we tested whether conspiracy spillover follows a similar trajectory in Twitter communication. We used a network topology approach to measure hashtag co-occurrences of the country hashtags in online communication (see STAR Methods).

As mentioned above, we used geospecific hashtags (#usa, #uk, #india, and #sweden) to study the contexts of SG conspiracy spillover in these countries. We chose the USA and the UK due to their strong global leadership in successfully conceptualizing and launching SG research projects (Figure 1). Sweden recently encountered massive public protests over SG deployment, demonstrating an interesting case of climate action. India has historically used geoengineering-driven measures for various activities, including drought management. Recently, the Indian federal government initiated several calls for international cooperation for SG research and development^43^ (see Figure S4). Thus, by selecting these countries, we analyzed geospecific online interactions on SG and evaluated the cross-section spillover of the chemtrails conspiracy theory.

**Figure 5:**
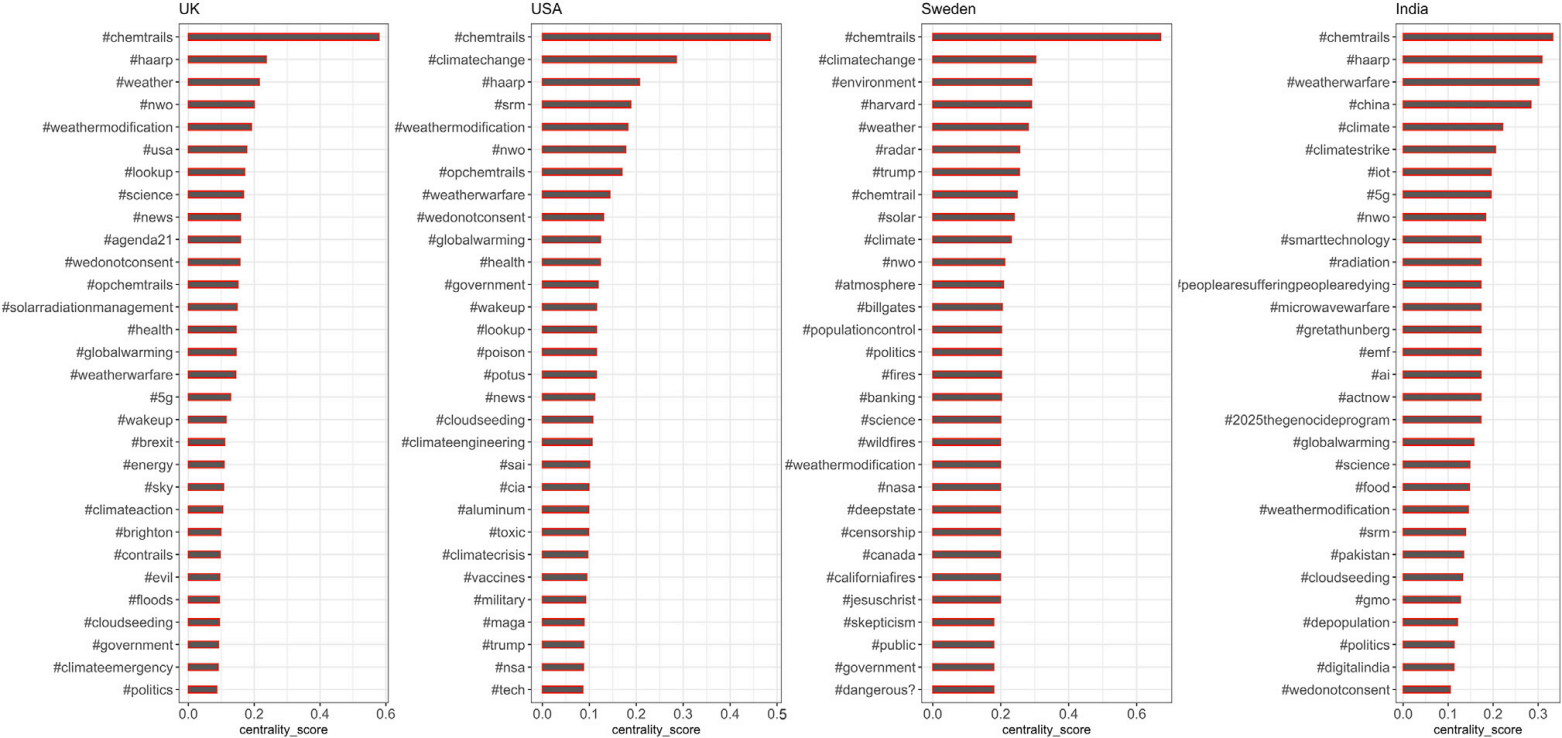
Influence of #chemtrails at the country-level hashtag co-occurrence network The x axis represents eigenvector centrality scores classified as low (0–0.09), mid (0.1–0.29), and high (0.3–1.0).

The first observation is that users who use #usa (n = 81,310) and #uk (n = 41,386) had much higher online social media engagement between 2009 and 2021 on #geoengineering than #sweden (n = 3,691) and #india (n = 2,689) (see Figure 5). This is at least partly driven by the fact that we only analyze English-language tweets. Using Twitter geolocation metadata, we found that ∼ 30% of the users who engaged with #usa were located in the USA. Around 55% of Twitter users engaging with the #uk were in the UK.

Similarly, almost 13% of the #india users had geolocation based in India. However, only ∼ 5% of #sweden users were geolocated to Sweden, implying that most of the country-specific online communication in our dataset is driven by USA and UK users.

The embedded emotional characteristics and the toxicity attributes of the specific tweets concerning national SG projects and governance events are provided in Figure S4.

SCoPEx was particularly important in Sweden since it was the project’s experimental test site.After the reports of its cancellation due to public outcry (in May 2021), emotion shifted from negative to positive (see Figure S4C). This reinforces our finding that social media interactions are reactive to such events. Local information can be readily accessed globally, influencing broader climate action narratives.

Results suggest that online reactions can be affected by the actors (like government funding bodies) and the declared purpose of the project (like drought management). Online toxicity analysis shows anti-SG narratives connecting the involvement of Bill Gates, the military, and chemtrails conspiracies across the four country hashtags. Interestingly, users in India and Sweden strongly emphasize SG as a “western influence” (see Table 2), indicating a spillover effect in online perceptions. Geospecific toxicity scores are presented in Figure S4E.

**Table 2:** Tweets with highest toxicity scores across the country scale

Toxicity score	Original Tweet
#UK	

0.98	Stupid F∗∗king idea is F∗∗king stupid. F∗∗k off Defra! UK smog warning my arse, u mean planes are coming to spray the sky with chemicals#Chemtrails #HAARP #geoengineering Massive attack in Bristol, uk right now !not the F∗∗king band you morons#geoengineering But don’t feel alone as Chemtrails here in uk every other day ! So we are F∗∗ked too !#geoengineeringAwful #chemtrails in Cheshire, UK today :(I AM SO SICK OF THIS s∗∗t! #geoengineering LOOK UP people! More frickin Chems∗∗t being spayed over uk now ! #geoengineering F∗∗king up you and the planet !!!

#USA	

0.99	Stop F∗∗king spraying us you F∗∗king Nazi cu∗∗s! @BillGates @richardbranson @Bayer #geoengineering #wedonotconsent F∗∗king fools. You’re all guilty of killing us all because you’re too F∗∗king dumb. The weather is controlled. #Geoengineering RIP idiots#geoengineering lucky us, we get one day of summer a year now. No wonder we need vitamin D Stop the F∗∗king #chemtrails you bastards!#ActOnClimate #GeoEngineering @realDonaldTrump #climatechange is the F∗∗king jets spraying us all @EU_Commission full of s∗∗t

#Sweden	

0.95	These dumb F∗∗ks are going to kill all of us. Sweden Scraps Bill Gates’ Geoengineering Plot To Block The Sun. Evil Billionaire idiots plan to block out the Sun! Yet billionaire idiot multiple 100,000 sq ft houses jets etc. but sheep but be sacrifice for elites billionaire idiots nonsense This is the stupidest idea ever! Let’s artificially make the whole planet colder? WTF NO NO NO … what is wrong with you? Planned Harvard balloon test in Sweden stirs solar geoengineering unease. Sounds like the idiots protesting the harmless balloon geoengineering experiment in Sweden

#India	

0.96	Lady VP and India. Obama is with Africa and Indonesia. I saw Berkeley and Baylor Texas try to unleash crypto programs. You F∗∗kers all look like brainless pieces of s∗∗t. Then there is the world geoengineering problem. Wow. What a show. also i had to reload a save because india F∗∗king nuked everyone because they were mad about my aerosol geoengineering. @RahulGandhi Who gives a s∗∗t! Let Indians come back. Why are we serving interests of (((Western Thugs))) economically All bulls∗∗t. Our EVMs are rigged by (((Western Thugs))). Just like they propped Hitler etc for WW2This is because (((@RoyalFamily @USAirforce @USNavy @CIA))) want to attack Mumbai (and rest of India) My country is facing full onslaught dirty tricks of ’Roman Empire’. Mark of the beast, political sabotage, crimes social unrest, baking sabotage, and modern stuff like geoengineering for total murder of India. I have zero sympathy for your bulls∗∗t. Get lost. Photographers all over India, how about starting to post photos of the sky wherever u live everyday, for chemtrail

Note: ∗ added by the authors to mask explicit language.

We find that the #chemtrail is central to many Twitter geoengineering conversations (see Figure 5), denoted through the highest centrality scores in the country-specific networks: #uk (∼ 0.58), #usa (∼ 0.47), #sweden (∼ 0.68), and #india (∼ 0.37). In addition, the #chemtrail connects to broader political movements and their narratives.

**Figure 6:**
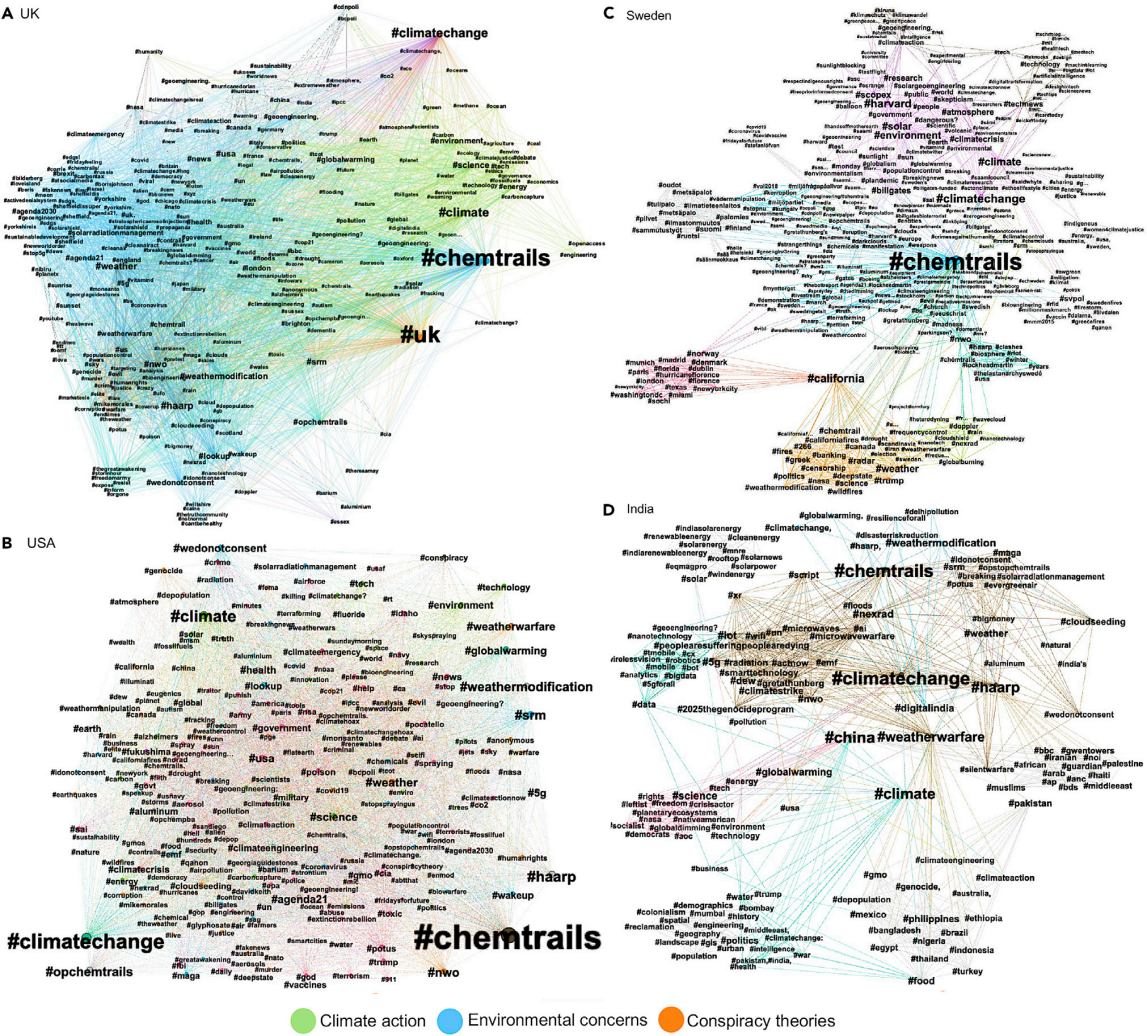
Hashtag co-occurrence networks showing spillover of #chemtrails conspiracy theory at the geospecific hashtag scale (A–D) #UK; (b) #USA; (c) #Sweden; (d) #India. The hashtag (node) size shows its influence in the respective networks, and the thickness of the lines (edges) represents the strength of connectivity, measured using eigenvector centrality. The color denotes respective hashtag clustering. Detailed network characteristics are shown in Table S5.

The UK-specific hashtag network has three distinct thematic clusters (see Figure 6A) with high centrality scores (> 0.4) connecting science and technology, concerns around weather modification, and strong opposition to SG. We also find moderate influence (centrality score 0.1–0.3) of conspiracy-related hashtags on public health, climate justice, and SG governance (Figure 5).

Unlike the UK hashtag co-occurrence network, the US results do not feature specific science- and technology-related hashtag clusters. Instead, we find clusters overlap more strongly, indicating a stronger integration of the conspiracy-related part of the geoengineering discussion into other parts. The influence of Donald Trump’s Deep State conspiracies^44^^,^^45^(#maga, #deepsate, #nwo, and #greatawakening) on the US #chemtrail network is strong (centrality score > 0.1). There are further spillovers to more recent conspiracy theories on Covid-19 vaccines^46^ (#antivax) and #qanon (Figures 5 and 6B). Bill Gates’s involvement with the SCoPEx project discussed earlier) can also be seen in this and the Swedish network (Figure 6C).

The Swedish #chemtrail network is interesting as a large subset of tweets comes from the US, primarily related to the SCoPEx project (Figure 5C). It also shows a strong presence of #trump and associated “deepstate” conspiracy hashtags, supporting our hypothesis of conspiracy spillovers.

Similarly, the Indian #chemtrail co-occurrence network has a stronger local geopolitical influence (centrality score = 0.1–0.3) shaped by #china, #pakistan, and #weatherwarfare, which connects SG conspiracy clusters. We also find presence of 5G conspiracy (#microwavewarfare, #radiation, and #2025thgenocideprogram) linking with #digitalindia movement (supporting^46^^,^^47^)(see Figure 6D).

Thus, we find that the SG debate on Twitter is dominated by conspiracy theories and leverages local political tensions to amplify its contagion and create a spillover effect (supporting the conclusion of^23^^,^^28^^,^^46^^,^^48^^,^^49^). A common observation across the four countries is the presence of #politics and #governance in SG conspiracy tweets indicating significant political influence and interests.

## Discussion

We examined public perceptions of solar geoengineering (SG) using a vast Twitter interaction dataset over 13 years. Social media can provide valuable information on how controversial climate action topics such as SG propagate online. At the same time, we show that perception is shaped and gets shaped by the local political context.

The strong connection to conspiracy theories and the presence of audiences that share this misinformation (i.e. causing a spillover) is extremely salient, as it shows that apparent connections to diverse conspiracy groups actively influence knowledge of SG. This bodes ill for future research because one cannot easily refute beliefs and the feelings that such misinformation campaigns perpetuate. This finding further reinforces the global call for transparent and public-facing SG research.^3^^,^^4^^,^^6^^,^^50^

The risk of conspiracy spillovers also raises concerns over inferring public attitudes from social media interactions, given that they appear to be so prone to manipulation, toxicity, or false information. This could have a grave consequence for SG research and governance since it can increase the public’s and policymakers’ skepticism and impede their ability to judge and act objectively.^51^^,^^52^

Moreover, we find positive emotions rise following SG governance events on global and country-specific scales. Conversely, negative and neutral emotions increase following SG projects and experiment announcements. Our findings suggest that negative emotions related to SG have a contagion effect, transcending regional boundaries and engaging with wider conspiracies (supporting^23^^,^^24^). For example, the influence of Trump’s “deep state” conspiracy was seen across the UK, India, and Sweden (Figure 6) while interacting with messaging on regional considerations (like Pakistan - China warfare for India, Brexit for the UK, and Bill Gates Dimming the Sun controversy for Sweden).

We also find that an increase in online engagement increases both negative emotions and toxicity attributes of the tweets (see Figures S2C and S4E). The narratives around high toxicity scores (Tables 1 and 2) show a distinct and strong influence of the chemtrails conspiracy theory and SG-science deniers. The implication is that we need more transparent SG research to clarify misconceptions and public engagement to limit toxicity spillovers. We found that conspiracy theories and political tensions directly influence online toxicity.

Social media analyses, tweet volume, and polarity shifts in emotion after major policy events (Figure 1) demonstrate that users are sensitive to how these events are communicated. Our results showed statistically significant increases in negative emotions and increases in Twitter engagement following important events ($R^{2}=10.02$, 99% CI, see Figure S1). For example, the reports of the SCoPEx project demonstrated a notable polarity shift from positive to negative with a significant increase in daily tweet volume (an ∼ 300% increase relative to February 2017 levels). Moreover, the social media influence of even relatively small NGOs demonstrated the mobilizing capabilities of social media platforms, for example, with the ETC Group and HOME campaign in Sweden helping to stop the SCoPEx launch (Figure S4C). Eventhough, Swedish government was the actor that ordered Swedish Space Corporation not to honor launch contract. Therefore, this demonstrates an opportunity for wider stakeholder engagement using a communication medium that can foster consensus-building dialogs with the public, experts, and other critical actors. This approach to making SG research more readily visible in the public domain through enhanced engagement was also emphasized by the NASEM consensus report,^3^ and our findings help inform how this might be achieved.

Furthermore, our country-scale spillover analysis (Figures 5 and 6) highlighted two critical findings. Misinformation campaigns and conspiracy theories do not always prevail; they will be filtered by local culture and institutions, social norms, and political dynamics.^53^ However, a cautionary implication is that no one-size-fits-all solutions exist; contextualized policy and governance design are needed to improve trustworthiness in climate action.

This paper does have certain limitations (details in the supplemental information). The Twitter dataset potentially embeds certain unintentional biases associated with its user demographics.^54^ Biases in the dataset also include manipulation of specific topics to trends to generate fake news and misinformation.^55^ Hashtags also include self-selection bias which can be resolved through nationally represented sample surveys.^56^ The scope of this paper was limited to a broad observational analysis of the #geoengineering, which we found to be heavily dominated by the chemtrails controversy with little engagement of the more legitimate scientific concerns of environmental scientists over SRM. Future work could study the discourse on genuine scientific controversies on social media platforms — in particular, how users react and discuss these topics. The link between the cause of online polarization and its effect on the spread of misinformation and conspiracy theories remains an active area of research.

Combining social media opinions with representative public opinion remains a future work of this study. Furthermore, our country-level tweet decomposition was limited in scope to users’ co-occurrences of geospecific hashtags. We found at least 80% of the tweets on #geoengineering originated from users with locations as “USA/United States of America/America/us/US/states/united states”. As it is difficult to geolocate all of the Twitter accounts in our dataset, we used country-specific hashtags to identify how users tweet about SG events or issues arising in these countries, thereby shaping our interpretation of the spillover effect. Another limitation is that the lexicon-based emotion analysis fails to capture more complex emotions and expressions like irony, sarcasm, etc. The estimated toxicity attributes’ accuracy also depends on its training data and modeling parameters set by Google.

Follow-up research should investigate which user groups engage in geoengineering debates to inform policymakers about the role of states, intergovernmental organizations, funding agencies, experts, and conspiracy theorists. It would also be interesting to explore how such debates shape policy and how different actor coalitions might benefit from misinformation campaigns.

Despite the scoping limitation, views expressed through Twitter do not necessarily translate directly into wider public views. We found that there is still a substantial value as large numbers of voices are being heard compared with other source types of opinion, such as focus groups. Moreover, as a data source, it showed the potential for a significant number of people to be exposed to an exchange of views or discussion on geoengineering (and its conspiracies) involving a fairly larger number of Twitter users. Therefore, establishing a future scope of work on how activities “outside” Twitter effects user reactions on the platform.

Nevertheless, our research fills a critical gap by broadening the understanding of online communications surrounding SG. There are opportunities to slow the spread of misinformation and conspiracy spillovers by moderating online toxicity on such topics, enabling greater online engagement with verified content, trustworthiness in climate action, and detangling sincere SG research efforts from the conspiracy-driven SG antithesis. We offer a way forward to understanding the structure of online climate misinformation through spillover effects, which can be used to create safer and better social media environments.

## Ethics declaration

The Institutional Review Board (IRB) reviewed this research at the Judge Business School, University of Cambridge (20–064) and the California Institute of Technology (21–1169). Twitter and Google were informed about this research during the v2API and Perspective API requests, respectively.

University IRBs are trusted with the review and approval of research like that reported in this paper, where data are collected and analyzed from human subjects. In this case, when an individual signs up for an account on Twitter, they are informed that what they post on the platform will be public information unless they make their activity private. Thus, users of the Twitter service know that what they post is public information. Twitter makes available to approved academic researchers various endpoints to collect different types of data, and academic use of the data that are collected from these endpoints is governed by terms of use for academic research.

In our use of these data, we use industry-standard methods to secure our data and restrict the use of the data to only the research team. We also avoid publishing any identifying information, like user names, when we produce research papers or other products describing our studies. To that end, we typically include in our studies mainly statistical aggregations and summaries that help mask the identities of the accounts that are in our datasets. Following Twitter’s academic terms of use, we can only share with other researchers limited information from our datasets, in particular the tweet identification numbers, which allow other approved academic researchers the information necessary to reconstruct our dataset.

Given that the data that we collect are public observation of behavior, that we collect data from the Twitter endpoints subject to the academic terms of service, and that we take a number of steps to secure the data and protect the identity of the users who provide the data for our analysis, there is only minimal risks for the users whose data factor into our research. Thus, the IRBs at our universities approved the research protocols for the collection and analysis of the data we use for this study as our data collection and analytic strategies are consistent with the principals in the Menlo Protocol.^57^

## STAR★Methods

### Key resources table

**Table:** 

REAGENT or RESOURCE	SOURCE	IDENTIFIER
**Software and algorithms**		

	Gephi v.0.9.2	https://gephi.org/
	R studio 2022–12.0.0-353	https://posit.co/
	GitHub	https://github.com/Ramit1201/geoeng

**Deposited data**		

	Mendeley Data	https://doi.org/10.17632/546hsym93p.1

### Resource availability

#### Lead contact

Further information and requests for resources and reagents should be directed to and will be fulfilled by the lead contact, Dr Ramit Debnath, rd545@cam.ac.uk/ramit.debnath@gmail.com.

#### Materials availability

This study did not generate new unique reagents.

### Method details

This section provides a detailed description of our data-driven approach to analyzing our diachronic Twitter dataset. It contains tweets, retweets, and shares between January 2009 to December 2021, where the hashtag #geoengineering was mentioned. At present, the growing field of computational social science warrants the use of social media big data (like Twitter datasets) that embeds individual-level user behavioral patterns.^58^^,^^59^ Twitter data provides information on public opinion about environmental issues and climate policies as it enables a diverse and world-wide range of user-generated content like texts, images, audio, video and live conversation strings.^58^ In this paper, we use natural language processing (NLP) to analyze the patterns in the text of each tweet’s “status update”. NLP is an interdisciplinary field that spans linguistics, computer science, deep learning and artificial intelligence (AI), enabling computers to “understand” human text and spoken words and the nuances of their embedded contexts and sentiments.

Our analysis is structured in four parts: First, we evaluate the online narrative regarding global policies and major solar geoengineering (SG) events like project announcements, experimental launches, governance, and policy negotiation events. We divided the observed time frame into three intervals: 2009–2012, 2013–2017 and 2018–2021. Second, we apply NLP methods to all 814,924 Tweets to extract the emotions and users’ perceptions of significant events. Findings are compared on a global and country scale (UK, USA, Sweden and India). We used lexicon-based emotion analysis (NRClex), word embeddings in chemtrails conspiracy theory, and hashtag extraction to detect how chemtrails connect with other conspiracy theories. The analysis of word embedding develops a representation of a word in a real-valued vector space that encodes the meaning in such a way that words closer in the vector space are expected to be similar. It places similar-meaning words closer to each other. This approach aided in mapping words closely associated with the chemtrails conspiracy theory in this study.

Third, we used deep-learning-based Google’s Perspective API to estimate toxicity attributes in the tweets to evaluate the public perception and the nature of conspiracy spillover over time. Google’s Jigsaw built this deep-learning and NLP-based platform to identify abusive content through the Perspective API (https://perspectiveapi.com/). In this paper, we use it to determine the toxic effect of solar geoengineering-related conspiracy theories on the Twitter conversation. Online toxicity poses a severe challenge to free speech, as toxic conversation and behaviors online can breed harmful echo chambers and aid in the dissemination of conspiracy theory.^59^ For example, online abuse and harassment silence important voices in conversation, forcing already marginalized people offline.^60^

Fourth, using network analysis, we investigate regional SG hashtag co-occurrence networks and their spillover effects at a global and country-specific scale. Network analysis examines the relationship among entities. This paper measures how SG hashtags are interconnected and their collective impact on the online Twitter discourse. We call these interconnections the spillover effect. Details of methodological steps are presented below.

#### Public twitter interactions as a data source

Twitter is one of the most popular social media platforms, with over 206 million daily users world-wide. Dubbed a ’digital townhouse’, it is an effective source of multidimensional information on public opinion on climate action, politics, technology, and economy.^61^^,^^62^ Twitter data offers a new cross-sectional advantage over current socio-economic survey data by capturing echoes of public opinion and emotions at a large scale.^61^^,^^63^^,^^64^

This paper uses Twitter’s latest v2 Application Programming Interface (API) to access the data. We used the R-programming language for the API access using the academictwitteR v0.3.0 package. As a search query, we specifically used the #geoengineering to capture any available tweets in the public Twitter domain during this 13-year time frame, collecting only English-language posts without any geographical restrictions. This produced a dataset of 814,924 tweets and retweets containing the above hashtags. We kept the search query as broad as possible to capture the most considerable bandwidth of public interactions with hashtags on geoengineering in the current climate change social media communication. In addition, we added #uk; #us, #usa; #india and #sweden using the ‘AND’ logical parameter to the initial search query for the country-specific analysis.

We assumed that with such country-specific hashtags, we could map the regional network overlapping characteristics of the tweets. For example, a tweet from a US author using #sweden would be included in both the US and Sweden networks. It was done to evaluate our conceptualised conspiracy spillover effects. Hashtags are a critical communication instrument for information propaganda on Twitter.^55^ Users deploy hashtags to annotate the content they produce, allowing other users to discover their tweets and enable network interactions on the platform.^65^^,^^66^^,^^67^^,^^68^^,^^69^ These characteristics of Twitter data made it a viable candidate for conspiracy spillover and public emotion analysis. Descriptive statistics are provided in Table S1.

#### Contextualised emotion analysis and its temporal shifts over time

The Twitter text data was processed using an NLP workflow using the tidyverse v1.3.1 and tidytext v0.3.2 packages in R. The workflow consisted of text preprocessing, feature extraction for n-grams and emotion analysis. The pre-processing stage consisted of tokenisation, stemming and lemmatisation (for the definition of these terms, see^70^). In this pre-processing stage, we removed the stopwords (see definitions^70^) in the data frame using the tm v0.7–8 package in R. This workflow extracted the cleaned base form of words from the tweets and generated the document-term-matrix (dtm) needed for emotion analysis.

In the next stage, we isolated individual hashtags from the data corpus. The hashtags were extracted from each tweet using a feature extraction-like data pipeline using the tidygramr v0.1.0 package to prepare n-gram models. Then, we extracted hashtag unigrams from each tweet and stored it as a separate dtm that included feature vectors of #ngram and the number of times it is repeated in an individual tweet (called ‘freq’). Both ‘#ngram’ and ‘freq’ were later used to create the hashtag co-occurrence network graphs. During this hashtag unigram feature selection process, we also ensured to exclude ‘#geoengineering’ (for the global analysis) and the respective country hashtags (#uk; #us, #usa; #india and #sweden) to reduce over-representation biases in our dtm.

We used NRC Word-Emotion Association Lexicon^71^ for the emotion analysis of the tweets using syuzhet v1.0.6 package. It consists of a list of English words and their connotations with the following emotions (anger, fear, anticipation, trust, surprise, sadness, joy, optimism, and disgust); the list of corresponding words/terms to the specific emotions can be found here.^72^

We add a methodological innovation here by categorising the NRC lexicon-driven emotion analysis by contextualising existing concerns, skepticism, oppositions, disagreements and controversies around SG that already have a strong negative stance (^4^^,^^16^^,^^25^^,^^31^^,^^73^^,^^74^^,^^75^). Thereby adding a supervised classification layer for domain adaptation.^76^^,^^77^^,^^78^^,^^79^^,^^80^ We make an assumption based on this general public stance on SG and manually categorise and regrouped the lexicon-derived emotions as positive (joy, trust and optimism), negative (disgust, fear, anger and sadness) and neutral (anticipation and surprise) emotions (also by^81^). This domain adaptation was performed to strengthen this study’s ’reactive analysis’ focus.

#### Estimating conspiracy embedding in tweets over time

We deconstructed the semantic structure of SG-conspiracy tweets using the word2vec word embedding technique and skipgram model.^82^^,^^83^ It is a technique for identifying similarities between words in a corpus, and it is widely used in automated text analysis to identify analogies.^84^^,^^85^ Word embeddings are created by identifying the words that occur within a “Context Window” - defined by a string of words before and after a “centre” word.^86^^,^^87^ The centre word and context words are represented as a vector of numbers (word2vec) to evaluate the presence or absence of unique words in the dataset. We use word2vec v0.3.4 in R to perform training and computations.

First, we filtered all retweets to avoid the creation of identical word vectors in our model that will become computationally expensive and produce poor results. Following this stage, we create a unique identifier for each tweet to facilitate tokenisation. Next, we use the skip-gram algorithm^82^^,^^83^ to estimate the probabilities of each word next to every other word within the context window. We define a context window of eight, i.e., we work in a batch of eight words in an individual tweet to estimate the conditional probability of other words (termed it as *p_together*) with the ’chemtrails’ conspiracy theory. A similar approach for investigating tweets through a context window was performed by.^88^^,^^89^

Once the *p_together* variable is estimated, we plot all of the words in our model in multidimensional space. It was done by creating a word embedding matrix, which generates a large matrix for each time frame (’2009–2012’, ’2013–2017’ and ’2018–2021’). We reduce the dimensions of this matrix using the Singular Value Decomposition (SVD) method using the irlba v2.3.4 package^90^ (see Figure S5).

#### Toxicity estimation as tweet attributes using deep learning

We used Google Jigsaw’s public Perspective API to estimate the level of toxicity and severe toxicity (as ’attributes’) in the Tweets. It is used to probabilistically score (0–1) the perceived impact of tweets on Twitter conversations with #geoengineering. A higher score indicated a greater likelihood that a reader/user would perceive the tweet as “Toxic” or “ Severely toxic”. Perspective API defines “Toxicity” as *’a rude*, *disrespectful or reasonable comment likely to make people leave a discussion’*.^91^ Similarly, “Severe toxicity” is defined as *’a very hateful*, *aggressive*, *disrespectful comment or otherwise very likely to make a user leave a discussion or give up on sharing their perspective*.^91^ We used the ’peRspective’ library v0.1.0 in R to interface with the Perspective API via the Google Cloud Platform.

Perspective’s architecture is based on multilingual deep-learning models like BERT (Bidirectional Encoder Representations from Transformers), trained from online forums such as Wikipedia (CC-BY-SA3 license) and the New York Times. The Perspective API provides pre-trained data based on its proprietary Perspective Pretraining Corpus (PPC) of ∼ 4.6 billion messages and comment texts.^92^ The BERT-based models were then distilled into single-language Convolution Neural Networks (CNNs) for each of the supported languages.^60^ Our paper relies solely on the English-language (en) models to analyze the Tweet corpus using this modelling approach.

We note that the accuracy of the estimated toxicity attributes is dependent on its dedicated pretrained corpus and modelling parameters. Google states that the model predicts higher toxicity scores for comments with terms for more frequently targeted groups (e.g., words like “black”, “Muslim”, “feminist”, “woman”, or “gay”) because comments about those groups are over-represented in abusive and toxic comments.^60^^,^^93^ We followed Google Jigsaw’s best practice guidelines to limit these biases in our analysis.^60^

#### Measurement of conspiracy theory spillover using network analysis

We extracted geospecific hashtags (as an #ngram) from the tweets to construct hashtag co-occurrence networks for #chemtrails across the UK, USA, India and Sweden. Co-occurrence networks are a graphical representation of how frequently variables appear to each other together.^94^ In our hashtag co-occurrence network construction, we measure how frequently (’freq’) two specific hashtags (#ngram) are presented in a single tweet. We used country-specific hashtags to identify how users tweet about SG events or issues arising in these countries, defining it as a “country-scale” analysis. A node represents each hashtag in the co-occurrence network, and the link between two nodes represents an edge, weighted by its frequency. We constructed undirected networks using the above nodes and edges description in Gephi 0.9.2. Moreover, to measure the influence of hashtags in these networks, we use eigenvector centrality scores, discussed below.

### Quantification and statistical analysis

We use statistical scaling of the derived emotion scores estimated from the tweets. It is scaled between 0 and 1 (feature scaled) through a min-max normalisation function (see Equation 1) in R to visualise its strengths across the Tweet time series (see Table S4). In addition, the time series of emotions were shown with their 6-month moving average (see Equation 2).

(Equation 1) $$normalized.values=\frac{value-minimum}{maximum-minimum}$$

(Equation 2) $$T_{t}=\frac{1}{m}\sum_{j=-k}^{k}y_{t}$$

where, m = 2k+1.The estimate of the trend-cycle at time *t* is obtained by averaging values of the time series within *k* periods of *t*.

Next, we evaluate the Spearman correlation between SG-driven Twitter engagement and its embedded positive and negative emotions. We report the following values for robustness and statistical significance: adjusted r-square, p-value, confidence interval and squared error terms. We also report the descriptive statistics associated with the Twitter interaction data, namely, retweet_count, like_count, user_tweet_count, user_following and user_follower (see supplemental information).

For the statistical transformation of the estimated word embeddings matrix from its higher dimensional space to lower dimensional space, we employ the Singular Value Decomposition (SVD) technique^95^(see Figure S4).

We used Perspective API’s state-of-the-art toxic content classifier for toxicity analysis, called UTC (Unified Toxic Content Classification). It leverages recent advances in Learnable Tokenizers, making it vocabulary- and token-free. This vocabulary-free property of UTC enables it to be both language agnostic and more robust to domain transfer.^92^^,^^96^ In line with the Perspective architecture, each comment/tweet was tagged by 3 to 10 crowdsourced raters and tagged whether or not a comment contained attributes of our interest (toxicity and severe toxicity). Detailed rater instructions can be referred to here.^97^ The English language (en) model performance scores (AUC-ROC) are 0.97 for toxicity and 0.98 for severe toxicity attributes (details here^60^). The descriptive statistics of the toxicity attributes are provided in Tables S2 and S4.

In network analysis of the conceptualised SG conspiracy spillover in the tweet hashtag network, a centrality measure called eigenvector centrality was used. The eigenvector centrality measures the influence of a node in a network and also evaluates a node’s importance while considering the importance of its neighbours.^98^ It is based on the principle that links from important nodes are valued more than links from trivial nodes. All nodes start equal; however, nodes with more edges start gaining importance as the computation progresses. Their importance propagates out to the nodes to which they are connected. Through iterative computing, the values stabilise, resulting in the final values for eigenvector centrality.^99^ Network characteristics are presented in Table S5 and Figure S6.

The derived graphs were optimised using the ForceAtlas2 (FA2) algorithm based on a force-directed layout that simulates a physical system to spatialise a network.^100^ The refinement of the network visualisation was performed by using various overlapping prevention layout settings in Gephi for the FA2 algorithm [A detailed mathematical background for FA2 is provided by Jacomy et al.^100^].

## Data Availability

• All data have been deposited at Mendeley Data and are publicly available as of publication. DOI is listed in the key resources table. Any additional information required to reanalyze the data reported in this paper is available from the lead contact upon request. • All codes have been deposited at GitHub and are publicly available as of the publication date. DOI is listed in the key resources table. Any additional information required to reanalyze the data reported in this paper is available from the lead contact upon request.
