# Supplementary material for: Conspiracy spillovers and geoengineering
Source: iScience. 2023 Feb 28;26(3):106166. doi: 10.1016/j.isci.2023.106166 (PMC10040962; doi:10.1016/j.isci.2023.106166)
Supplement: Document S1. Figures S1–S6 and Tables S1–S5 [file mmc1.pdf]

iScience, Volume *26*

Supplemental information

Conspiracy spillovers and geoengineering

Ramit Debnath, David M. Reiner, Benjamin K. Sovacool, Finn Müller-Hansen, Tim Repke, R. Michael Alvarez, and Shaun D. Fitzgerald

Supplementary Information (SI):
Conspiracy spillovers and geoengineering

Ramit Debnath, David M. Reiner, Benjamin K. Sovacool, Finn Muller-Hansen, Tim Repke, R. Michael Alvarez, and Shaun Fitzgerald

The descriptive characteristics of the #geoengineering tweets is illustrated in SI Table1.

SI Table 1: Descriptive characteristics of the Tweet corpus (n = 814, 924), related to STAR Methods

User_metrics	Minimum	Median	Mean	Maximum
Retweet_count	0	1	12.70	2029
Like_count	0	0	0.45	2631
User_tweet_count	1	44479	184712	3980074
User_Followers	0	1895	4539	19370201
User_Followings	0	1694	3016	925922

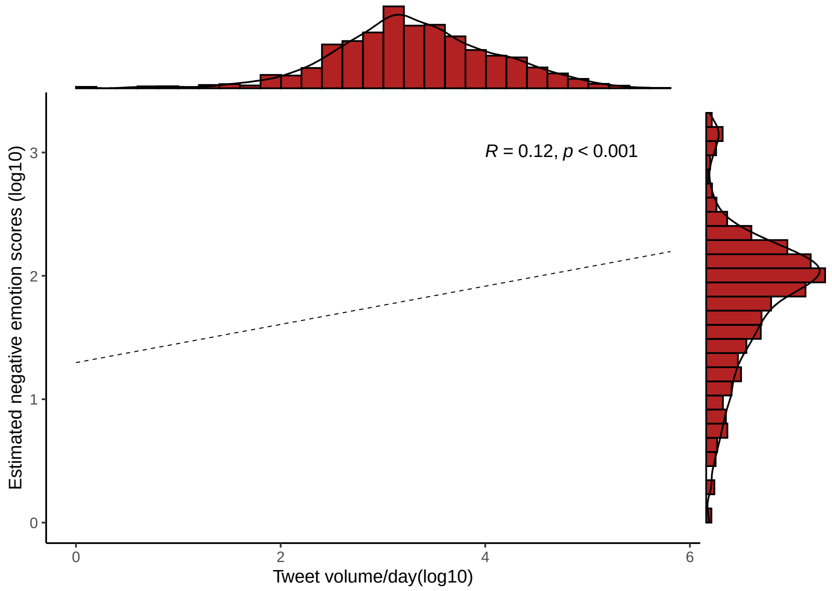

SI Figure 1: Spearman correlation between negative emotion and daily tweet volume of #geoengineering (n = 814, 924). The adjusted R-squared value is 0.1002, standard-error is 0.01920 significant at 0.001 level. [Related to Figure 1]

SI Figure 2 shows that severe toxicity is distinctively right-skewed with a single peak, with a 13-year mean score of 0.12 and a median score of 0.07. The toxicity attribute has multiple peaks with a rightward skew, with a 13-year mean score of 0.17 and a median score of 0.11. Furthermore, our approach classified ~60% of tweets as toxic relative to severe toxic between February 2009 and August 2021 (see SI Figure 2b). The share of severe toxic tweets further fell by 62.5% from August 2021, which coincides with the IPCC AR6 announcements on the assessment of solar geoengineering. The descriptive statistics details of the toxicity attributes are presented in SI Table 2.

SI Table 2: Descriptive characteristics of the toxicity attributes in the Tweet corpus, related to Figure 3

Attributes	Minimum	Median	Mean	Maximum
TOXICITY	0	0.13	0.17	0.99
SEVERE_TOXICITY	0	0.08	0.12	0.99

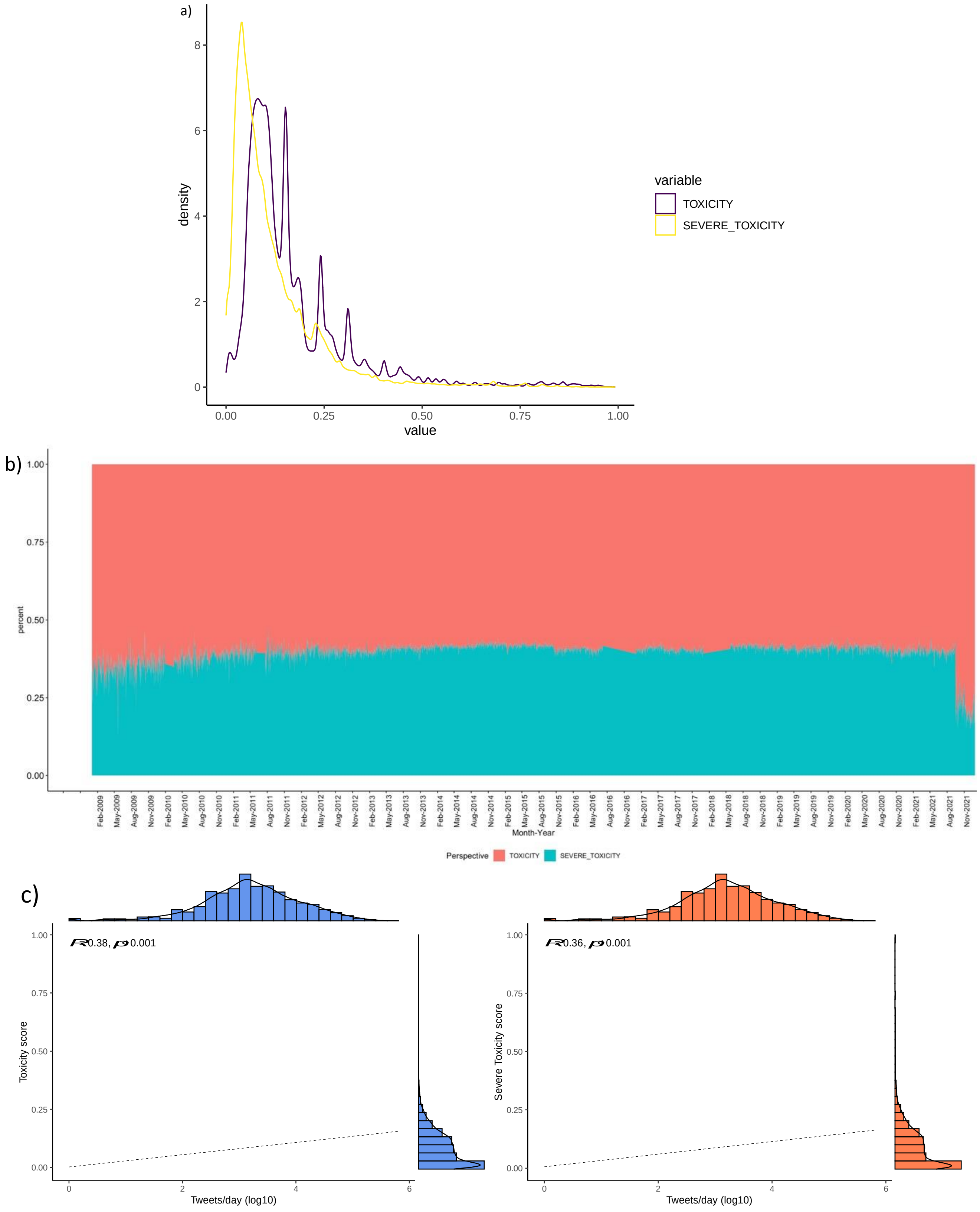

SI Figure 2: (a) Density characteristics of TOXICITY and SEVERE_TOXICITY estimates; (b) Temporal shifts in share of the TOXICITY and SEVERE_TOXICITY scores over the 13-year period; (c) Spearman correlation between tweets per day, TOXICITY (adjusted R-square = 0.0812, std. error = 0.0013) and SEVERE_TOXICITY (adjusted R-square = 0.0719, std. error = 0.0014), significant at 0.01 level. [Related to Figure 3 and Table 1]

We show in SI Figure 3 that the UK and the USA labelled tweets have similar toxicity distributions, with similar right skewness. The distribution differs drastically for Sweden and India, with India demonstrating localised peaks in high TOXICITY (0.85) and SEVERE_TOXICITY (0.73) scores.

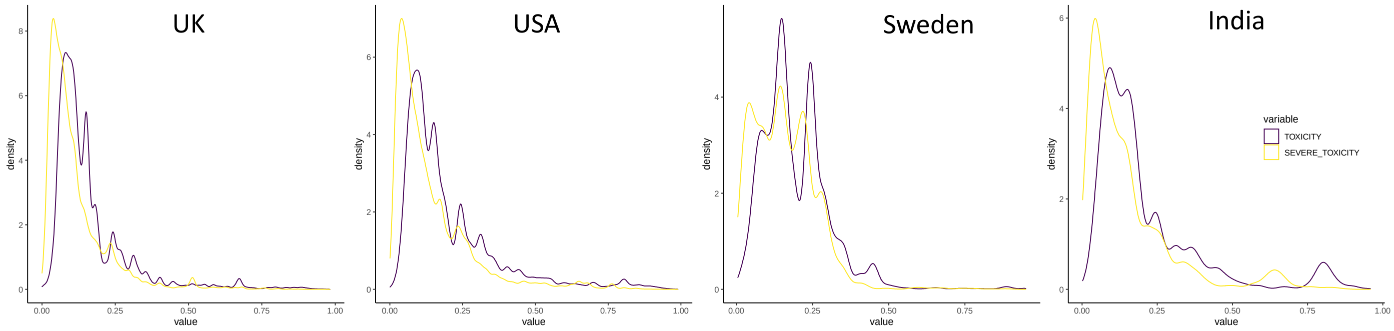

SI Figure 3: Density estimate characteristics of TOXICITY and SEVERE_TOXICITY by country label [Relate to Table 2].

SI Table 3: Descriptive characteristics of the toxicity attributes at the country scale, related to Table 2

Attributes	Minimum	Median	Mean	Maximum
TOXICITY (USA)	0.001	0.150	0.202	0.990
SEVERE_TOXICITY (USA)	0.000	0.094	0.139	0.941
TOXICITY (UK)	0.000	0.120	0.160	0.980
SEVERE_TOXICITY (UK)	0.000	0.008	0.110	0.920
TOXICITY (Sweden)	0.010	0.160	0.190	0.950
SEVERE_TOXICITY (Sweden)	0.010	0.150	0.160	0.890
TOXICITY (India)	0.000	0.150	0.210	0.960
SEVERE_TOXICITY (India)	0.000	0.100	0.150	0.870

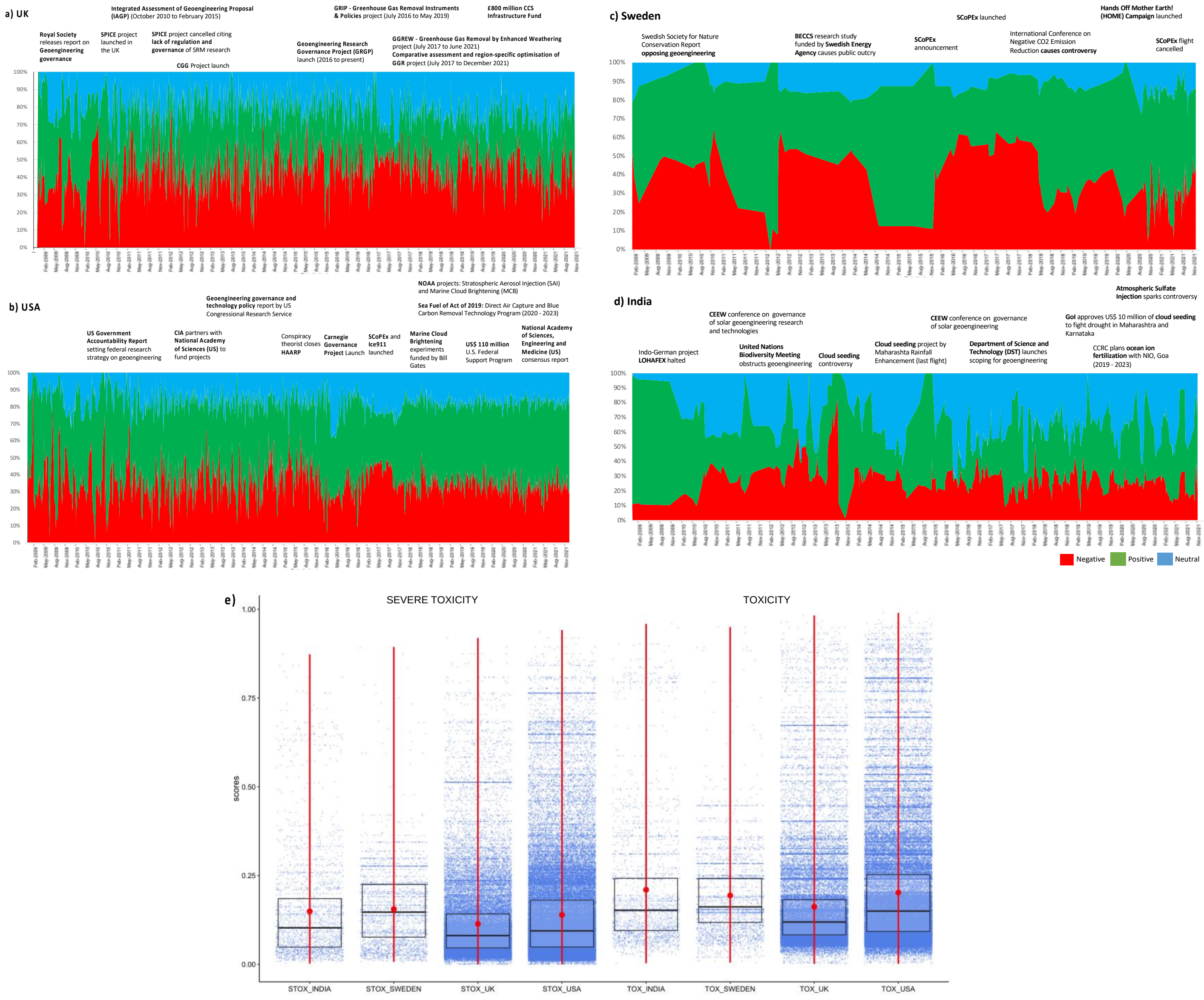

SI Figure 4: A 6-months moving-average representation of Twitter emotions associated with major SG projects and governance events for country hashtags between 2009-2021. The emotion scores are estimated using a lexicon-based approach (NRC-lexicon) with the following embedded emotions in each category: positive emotions (optimism, joy and trust), negative emotions (disgust, fear, anger and sadness) and neutral emotions (anticipation and surprise). The country hashtags are: (a) United Kingdom (UK) (n = 41,386), (b) United States of America (USA) (n = 81,310), (c) Sweden (n = 3,691), (d) India (n = 2,689). The red-dot shows the mean, and the red-line shows max and min values. [Related to Figure 5, Figure 6 and Table 2]

Tweets with UK hashtags have the highest share of negative and neutral emotions of the countries examined. For example, moving average estimation shows that neutral emotions such as surprise and anticipation increased (by more than 50%) in UK hashtagged communications when SG projects (like SPICE, GRGP and GRIP) were launched (SI Figure 4a).

Unlike the UK hashtagged tweets, the USA hashtagged have a consistently higher share of positive emotions across the timeline (SI Figure 4b). However, positive emotions (almost 40%) were more distinct for governance-related events like the launch of significant reports (SI Figure 4b). On the other hand, SG funding announcements and project launches were associated with a 25% rise in negative emotions (disgust, fear, anger and negative) (see SI Figure 4b).

The share of neutral emotions (anticipation and surprise) rose fourfold at the launch of the Carnegie Governance Project in May 2016 (see SI Figure 4b). By contrast, sharp peaks in negative (and neutral) emotions were observed at the launch of the gates-funded Marine Cloud Brightening experiments (August 2018). As previously noted, this association has attracted conspiracy theorists.

SG-related interactions on Twitter for Sweden-hashtagged tweets have a distinct cyclical pattern. Figure 4c shows that the share of negative emotions rises following various SG opposition and governance events. A prominent anti-solar geoengineering campaign called 'Hands Off Mother Earth! (HOME)' [1] (December 2020) created a nationwide public appeal to stop the SCoPEx launch. An open letter signed by authors in 45 countries contributed to abandoning the experiment. During this time, the share of negative emotions increased strongly, demonstrating the ability of social media to reflect and potentially shape public outcomes on SG events.

The Indian case is also interesting since the share of positive emotions in Indian hashtagged tweets gradually decreased over the 13 years (SI Figure 4d). Abrupt changes in emotion were tied to prominent national SG events. Notably, the cloud seeding controversy by non-state actors led to negative emotions rising more than threefold in August 2013 (SI Figure 4d). However, when state-based agencies sanctioned the same cloud seeding projects as drought management measures in May 2015, positive emotions increased sharply by almost 35% (see SI Figure 4d). A distinct feature of the Indian case is that many SG programs (especially cloud seeding) are operated by the federal government's Department of Science and Technology, which sets the overall governance agenda and is in favour of scientific research on SG [2].

SI Figure 4e show the characteristics of toxicity and severe toxicity in the tweet corpus across the four countries. Mean severe toxicity scores for India (0.15) and Sweden (0.16) are slightly higher than for the USA (0.14) and UK (0.11). For the toxicity scores India (0.21) remains highest, followed by the USA (0.20), Sweden (0.19), and the UK (0.16) (see SI Figure 4e and SI Table 3). Tweets with high toxicity attributes across the countries are illustrated in Table 2.

SI Table 4: Descriptive characteristics of the normalised emotion scores in the geospecific hashtags, related to Figure 5 and Figure 6

Emotions	Minimum	Median	Mean	Maximum
**#geoengineering**				
Positive	0	0.061	0.091	1
Negative	0	0.344	0.062	1
Neutral	0	0.023	0.051	1
**#UK**				
Positive	0	0.004	0.011	1
Negative	0	0.008	0.015	1
Neutral	0	0.004	0.003	1
**#USA**				
Positive	0	0.019	0.034	1
Negative	0	0.014	0.028	1
Neutral	0	0.006	0.015	1
**#INDIA**				
Positive	0	0.014	0.019	1
Negative	0	0.007	0.017	1
Neutral	0	0.018	0.027	1
**#SWEDEN**				
Positive	0	0.011	0.032	1

Negative	0	0.011	0.038	1
Neutral	0	0.010	0.031	1

SI Table 5: Network characteristics of the geospecific hashtag network, related to Figure 6

Country	Nodes	Edges	Mean weighted degree	Mean modularity class	Mean clustering coefficient	Mean eigenvector centrality score
UK	4581	24843	12.23	171.99	0.77	0.021
USA	7496	40359	9.80	337.31	0.41	0.013
India	418	1793	10.00	25.88	0.051	0.82
Sweden	668	3043	8.74	29.57	0.83	0.089

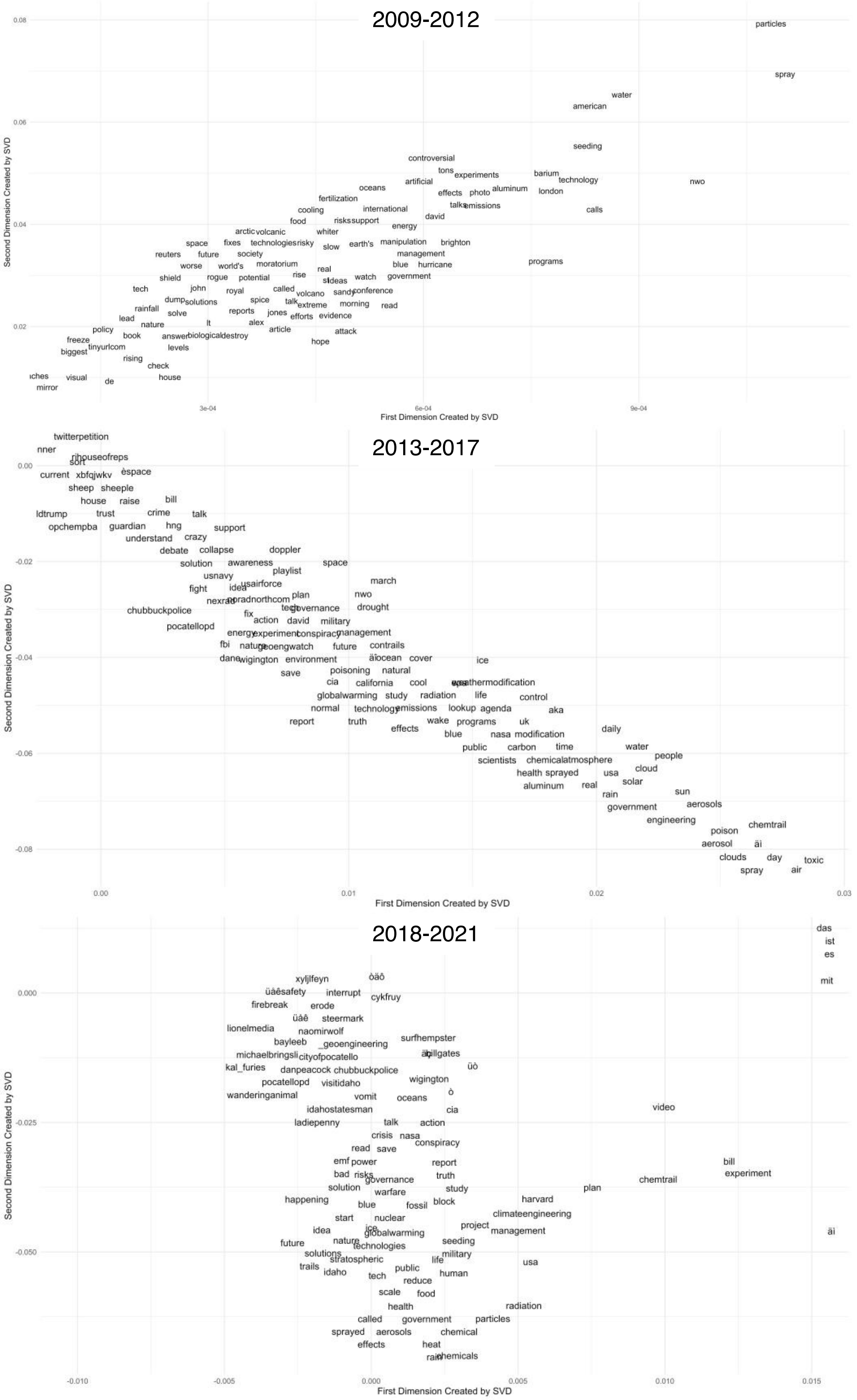

SI Figure 5: Dimension reduction embeddings of ‘chemtrails’ in the tweet corpus across the three-time scale, related Figure 3 and Figure 4.

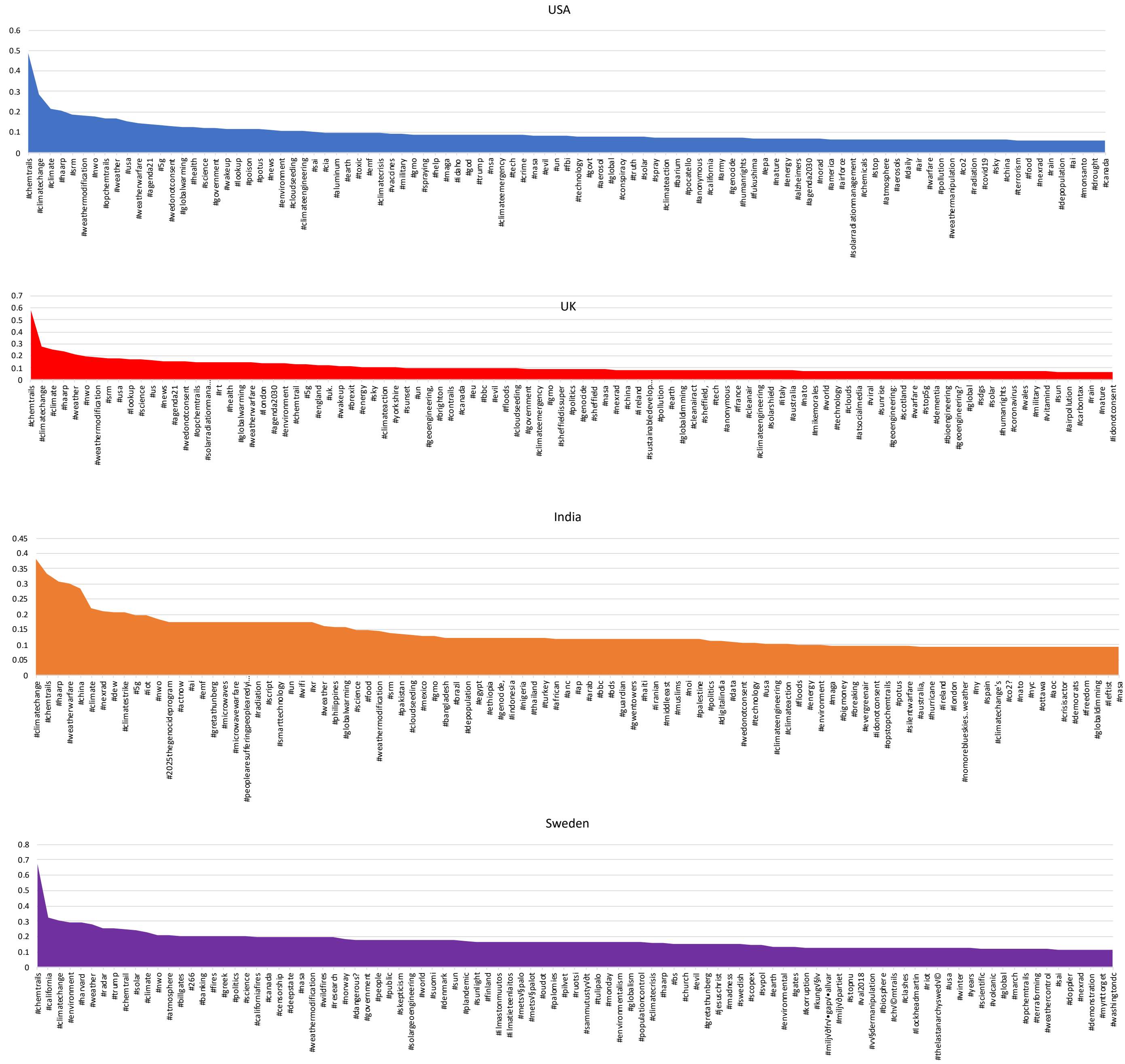

SI Figure 6: Top 100 hashtags with eigenvector centrality score, related to Figure 6 and STAR Methods.

References

1. HOME. *Hands off Mother Earth*, 2020. https://www.geoengineeringmonitor.org/wp-content/uploads/2018/10/home-new-EN-feb6.pdf

2. G. Bala and Akhilesh Gupta. Solar geoengineering research in India. *Bulletin of the American Meteorological Society*, 100(1):23–28, 2019. https://www.jstor.org/stable/26639248
